# Neuroinflammatory Consequences of Rhinovirus Infection in Human Epithelial and Neuronal Models

**DOI:** 10.1007/s00408-025-00846-y

**Published:** 2025-09-07

**Authors:** Orla M. Dunne, Nicola A. M. Roe, Aurélie Mousnier, S. Lorraine Martin, Gerard P. Sergeant, Imad About, Ikhlas El-Karim, Fionnuala T. Lundy, Lorcan P. McGarvey

**Affiliations:** 1 The Wellcome-Wolfson Institute for Experimental Medicine, School of Medicine, Dentistry and Biomedical Sciences, Queen’s University Belfast, 97 Lisburn Road, Belfast, Belfast BT9 7BL UK; 2 School of Pharmacy, Queen’s University Belfast, Belfast, UK; 3 Smooth Muscle Research Centre, Dundalk Institute of Technology, Dundalk, Co., Louth, Ireland; 4 Aix-Marseille Université, CNRS, ISM, Inst Movement Sci, Marseille, France

**Keywords:** Chronic cough, Cough hypersensitivity, Rhinovirus, TRPA1

## Abstract

**Introduction:**

Rhinovirus (RV) is the leading cause of exacerbations of lung disease. A sensory neuronal model, derived from human dental pulp stem cells and differentiated into peripheral neuronal equivalents (PNEs), was used to examine RV’s effects on airway sensory nerves. We investigated whether RV can directly infect and alter PNEs or whether it exerts effects indirectly via the release of mediators from infected epithelial cells.

**Methods:**

PNEs or primary bronchial epithelial cells (PBECs) were infected with the RV-A16 strain. Viral replication was confirmed by viral titration assays, immunofluorescence (IF) for the double-stranded RNA (dsRNA) replication intermediate and western blotting (WB). RNA sequencing was used to determine transcriptomic changes in PNEs, and inflammatory responses were assessed by inflammatory microarray. Calcium mobilisation assays were used to investigate the effect of interleukin-1β (IL-1β) on PNE transient receptor potential (TRP) A1 channel responses.

**Results:**

Viral titrations, WB and IF confirm RV-A16 entry and replication in PNEs and PBECs. Gene signatures associated with antiviral immune responses, sensory neuropathies and N-Methyl-D-aspartic acid (NMDA) receptor activity were upregulated in RV infected PNEs. Several cytokines were increased from PNEs and PBECs following RV infection, most notably IL-1β. Treatment of PNEs with IL-1β resulted in heightened TRPA1 channel sensitivity.

**Conclusion:**

We report the suitability of an airway neuronal model for the study of the direct effects of RV infection on nerves. RV-induced release of IL-1β from airway epithelium heightens neuronal TRPA1 responses suggesting a mechanism for virus-induced cough hypersensitivity.

**Supplementary Information:**

The online version contains supplementary material available at 10.1007/s00408-025-00846-y.

## Introduction

Rhinovirus (RV) infection is the leading cause of the common cold and frequently associated with exacerbations of asthma and chronic obstructive pulmonary disease (COPD) [1, 2]. Infected individuals typically report increased coughing triggered by relatively innocuous stimuli due to heightened cough reflex sensitivity [3, 4]. Although the mechanisms associated with human RV infection, in particular virus particle entry into the airway epithelium, viral replication and the ensuing inflammatory response are well described [5, 6], the interaction of this virus with airway nerves to induce heightened cough sensitivity is largely unknown.

Proposed mechanisms include the indirect effects of inflammatory mediators released from virally infected bronchial epithelial cells [7]. These factors can stimulate and potentially sensitise irritant receptors (nociceptors) on peripheral sensory afferent nerves by increasing cell expression and excitability of nociceptors and altering neuronal gene expression [8, 9].

Airway sensory nerves originate in vagal ganglia at the base of the skull with axons extending to sites beneath and within the airway epithelium [10–12]. Given such close proximity to the airway lumen, together with epithelial disruption which typically accompanies microbial infection [13], airway nerves are likely exposed to viral particles; an idea supported by a recent study demonstrating direct and acute activation of airway sensory nerve endings by a SARS-CoV-2 spike protein [14]. While some viruses have known neuropathic effects, the potential for RV to directly infect the respiratory nerves remains largely unstudied [15, 16].

Studying intact human airway sensory neurons is difficult because, as alluded to above, their cell bodies reside in extrapulmonary sites inaccessible via peripheral biopsy. Previous research into neural dysfunction associated with respiratory viral infection has relied on animal models or neuronally differentiated immortalised cell lines, which may not accurately represent in vivo human airway physiology [9, 17]. To address some of these limitations, we have used techniques first described by Arthur and colleagues [7, 8], and further adapted by our group [9, 10], to differentiate human adult dental pulp stem cells into an in vitro sensory neuronal model, termed ‘peripheral neuronal equivalents’ (PNEs). DPSCs are derived from the neural crest and thus share a developmental lineage with neural crest–derived sensory neurons, such as those in the jugular ganglion, but not with nodose neurons, which originate from epibranchial placodes. PNEs display morphological, molecular and functional characteristics of sensory neurons, express neuropeptides including substance P and CGRP, nociceptors such as transient receptor potential ankyrin receptor 1 (TRPA1) and have been used to explore mechanisms underlying chronic pain [18] and neural hyperresponsiveness to the viral mimetic polyinosinic:polycytidylic acid (poly I:C) [19]. This study is the first to examine the effects of infectious RV on PNEs.

The aim of this study was to provide evidence for the suitability of our PNE model to study RV associated cough reflex hypersensitivity. We investigated whether PNEs express the RV entry receptor ICAM-1 and hypothesised that RV directly enters and replicates in neuronal cells, leading to cytopathic effects (CPE) and inflammatory mediator release. As airway epithelial cells are the primary site for RV entry and replication in the lung, we used a primary bronchial epithelial cell model to investigate the indirect effect of a RV-induced airway epithelial inflammatory response on neuronal function.

## Materials and Methods

### Sensory Neuronal Model

A sensory neuronal model, in which stem cells were differentiated towards PNEs, was generated and maintained as previously described by our research group [18, 19]. Briefly, human dental pulp cells (hDPCs) were obtained from third molar teeth in compliance with French legislation and Aix-Marseille University ethical committee agreement (N/Ref: 2022-05-12-003). Dental pulp stem cells (DPSCs) were isolated from hDPCs using a fibronectin adhesion protocol. DPSCs were differentiated on culture ware coated with 0.01% poly-l-ornithine (Sigma Aldrich) and 5 μg/mL laminin (VWR International). Neurogenic media was composed of neurobasal-A media (Thermo Fisher Scientific) supplemented with, 1% GlutaMAX 100X (Thermo Fisher Scientific), 1% penicillin and streptomycin (Thermo Fisher Scientific), 1% B27 (Gibco, Life Technologies), 40 ng/mL basic fibro-blast growth factor (Peprotech Ltd) and 20 ng/mL epithelial growth factor (Peprotech Ltd) for 7–10 days.

Immunostaining of hDPCs, DPSCs and PNEs for fibroblastic surface protein (FSP) and neuronal marker PGP9.5 was carried out to characterise the neurogenic differentiation of DPSCs to PNEs.

### Primary Bronchial Epithelial Cell Culture

Primary bronchial epithelial cells (PBECs) were obtained from patients undergoing bronchoscopy for clinical purposes with ethical approval from the NHS Health Research Authority, East of England–Cambridge East Research Ethics Committee (REF 18/EE/0048). PBECs were cultured in PnuemoCult™ – Ex Plus medium (StemCell Technologies) supplemented with accompanying 50X supplement and hydrocortisone and used up to passage 5. A total of 4 PBEC samples (2 males and 2 females) were used throughout this study, with donor details available in supplementary Table 1.

### Infection of Cells with Rhinovirus

The RV strain RV-A16 was obtained from the American Type Culture Collection (ATCC VR-283) and was propagated and titrated in HeLa-H1 (ATCC CRL-1958) cells at 33 °C. Differentiated PNEs or PBECs were cultured in their respective supplement-free medium for 24 h before infection. PNEs or PBECs were infected with RV-A16 at a range of multiplicities of infection (MOI) [0.01 – 10] over 2 – 72 h. Control cells were mock-infected with culture medium alone or UV-inactivated virus. RV-A16 replication in PNEs and PBECs was analysed by viral titration, IF for the double-stranded RNA (dsRNA) replication intermediate, and western blotting for RV-A16 capsid proteins VP0/VP2 (R16-7 antibody, Covalab).

### RNA Sequencing of Rhinovirus Infected PNEs

PNEs were infected with RV-A16 (MOI 1) or mock-infected for 48 h. Cells were labelled with anti-dsRNA antibody (Scicons) and analysed by FACS. RNA was generated from the positive fraction of dsRNA labelled PNEs and mock-infected controls using the Maxwell simply cells kit (Promega). Library preparation was carried out with QuantSeq 3’ mRNA-Seq library prep kit (Lexogen) and sequenced on Miseq platform (Ilumina). Each condition (mock-infected and RV-A16 infected) was generated using 3 independent differentiations of DPSCs to PNEs that were pooled prior to the preparation of bulk RNA sequencing libraries. All genes with a Log twofold change > 2 were deemed upregulated. Functional enrichment analysis of upregulated genes was performed using ToppGene (gene ontology, human phenotype, disease categories) [20]. Reactome database was used for ingenuity pathway analysis (I.P.A) of upregulated genes [21].

### Investigation of TRPA1 Expression, Functionality and Hypersensitivity in PNEs

TRPA1 expression in PNEs was assessed by immunostaining. A Fura-2 AM (Thermo Fisher Scientific) assay was used to assess TRPA1 responses in PNEs as described in the online supplement. TRPA1 dose response curve to cinnamaldehyde [0.0001 to 1 µM] (Sigma Aldrich) in the absence and presence of the antagonist HC-030031 [50 µM] (Tocris) were evaluated. The effect of IL-1β [2 ng/mL] pretreatment on cinnamaldehyde responses [0.1 pM to 1000 µM] was also assessed by Fura-2 AM assay. The concentration of 2 ng/mL IL-1β was selected for use based on results previously published by Shi et al. demonstrating a similar concentration of IL-1b release from infected epithelial cells [22].

### Statistical Analysis

Data are presented as mean ± standard error of the mean (SEM) of at least 3 independent experiments, unless otherwise stated. Statistical comparisons were performed using Prism V9.3.1 (GraphPad Software) with P < 0.05 considered statistically significant. Data normality was assessed using the Shapiro–Wilk test. For normally distributed data, two-group comparisons were determined using a T-test, and for comparison of multiple treatments versus a single control a one-way ANOVA with a Dunnett’s post-hoc test was used. To analyse the interaction between multiple cinnamaldehyde concentrations in various conditions in Fura-2 AM assays a Two-way ANOVA with Šídák’s multiple comparisons test was used. Gene enrichment and I.P.A. analyses used hypergeometric testing with Benjamini–Hochberg correction.

Materials and methods are described in further detail in the online supplement.

## Results

### PNEs Display Neuronal Characteristics

**Fig. 1:**
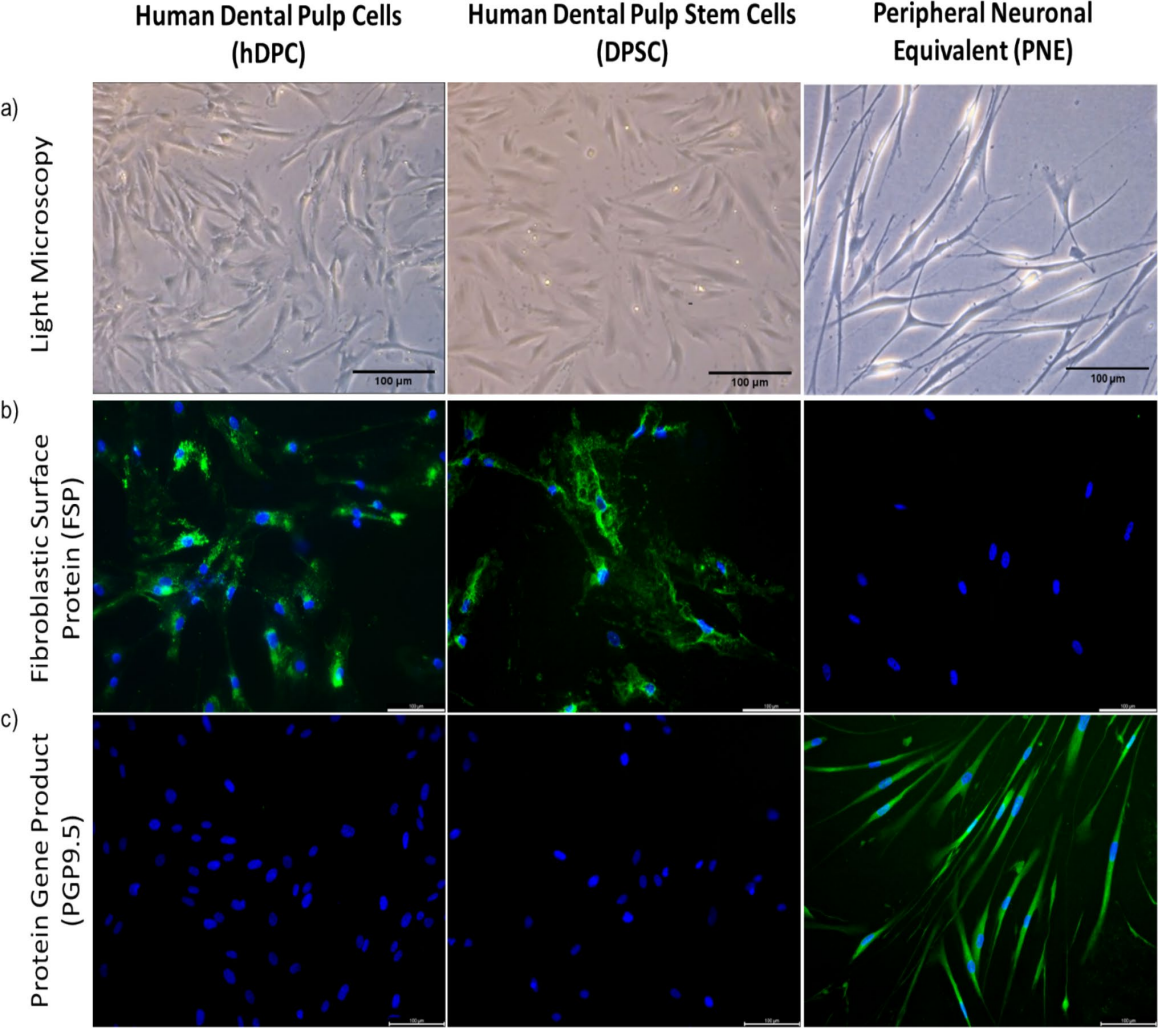
Changes in Morphology and Phenotype were Observed upon Human Dental Pulp Stem Cell Differentiation to Peripheral Nerve Equivalents. The morphology of human dental pulp cells (hDPC), dental pulp stem cells (DPSC) and peripheral nerve equivalents (PNEs) were observed using light microscopy **a**. Fibroblastic surface protein (FSP) expression was observed in hDPC and DPSCs but not in PNEs **b**. No expression of the neuronal marker protein gene product (PGP9.5) was observed in hDPC and DSPC but was expressed in PNEs **c**. Nuclear stain, DAPI (blue) in each immunofluorescent image (b & c). Scale bars 100 µm on all images

Following neurogenic differentiation, DPSCs underwent phenotypic shift from fibroblastic to neuronal morphology with a swollen cell body and axon-like projections (Fig. 1a).

The neuronal phenotype of the PNE model was verified using immunostaining. The fibroblastic marker, fibroblast surface protein (FSP), was highly expressed in both hDPCs and DPSC but the expression of this marker was lost following successful differentiation to PNEs (Fig. 1b). Expression of the neuronal marker PGP9.5 was absent in undifferentiated cells; however, PGP9.5 was highly expressed following neuronal differentiation to PNEs (Fig. 1c, negative controls in supplementary Fig. 1).

### Differentiated PNEs and PBECs are Susceptible to RV-A16 Infection and Permissive for its Reproduction

**Fig. 2:**
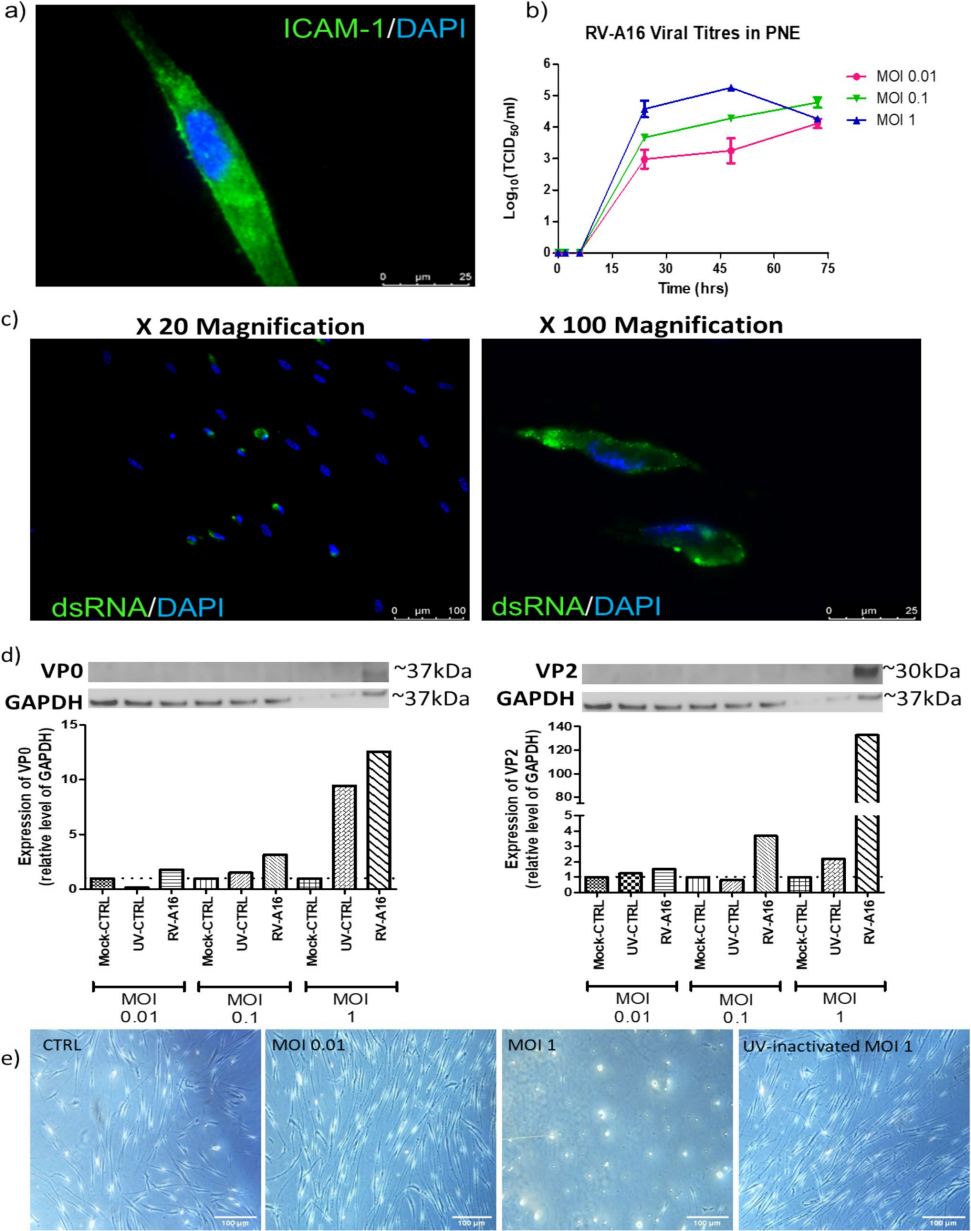
PNEs are Susceptible to RV-A16 Infection and Permissive for its Reproduction. Immunofluorescence demonstrates protein expression of intracellular adhesion molecule 1 (ICAM-1) in PNEs. Scale bars 25 µm **a**. Viral titrations demonstrate the production of new infectious viral particles in PNEs infected with RV-A16 at an MOI of 0.01, 0.1 and 1. TCID_50_ = 50% tissue culture infectious dose. Each point represents the mean ± SEM for 3 independent experiments **b**. Immunofluorescence demonstrates that dsRNA (green) is detected in PNEs infected for 48 h with RV-A16 (MOI 1), at 20 × magnification (scale bar 100 µm) and 100 × magnification (scale bar 25 µm) **c**. Western blots probed with R16-7 antibody show the presence of the viral capsid precursor VP0 (~ 37 kDa) and the mature viral capsid protein, VP2 (~ 30 kDa) in PNEs infected for 24 h at MOI 1. Semi-quantitative densitometry analysis of immunoreactive bands was carried out and expressed as fold change over GAPDH. N = 1 representative experiment **d**. Representative light microscopy images of PNEs infected with RV-A16 (MOI 0.01 or 1) at 48 h.p.i, with UV-inactivated control at MOI 1 (far right) or mock-infected control (far left) also presented. CPE in the form of cell rounding is visible in PNEs infected with RV-A16 at MOI 1 (centre right image). Scale bars 100 µm **e**

Positive immunostaining for ICAM-1 in PNEs, suggests susceptibility to infection by major group RV serotypes (Fig. 2a). Viral titration confirmed production of infectious RV progeny following infection of PNE with RV-A16 between 6 and 72 h.p.i (hours post-infection) (Fig. 2b), indicating that PNEs are both susceptible to RV-A16 infection and permissive for its reproduction. Positive immunostaining for the dsRNA viral replication intermediate was observed in PNEs infected for 48 h with RV-A16 (MOI 1), indicating active RV RNA replication (Fig. 2c and negative controls shown in supplementary Fig. 4). Both the viral capsid precursor protein and the mature viral capsid protein were detected by western blotting in PNEs infected for 24 h with RV-A16 at MOI 1 (Fig. 2d). CPE, defined as cell rounding and cell detachment from the culture vessel, was observed in PNEs 48 h.p.i at MOI 1 by light microscopy (Fig. 2e).

**Fig. 3:**
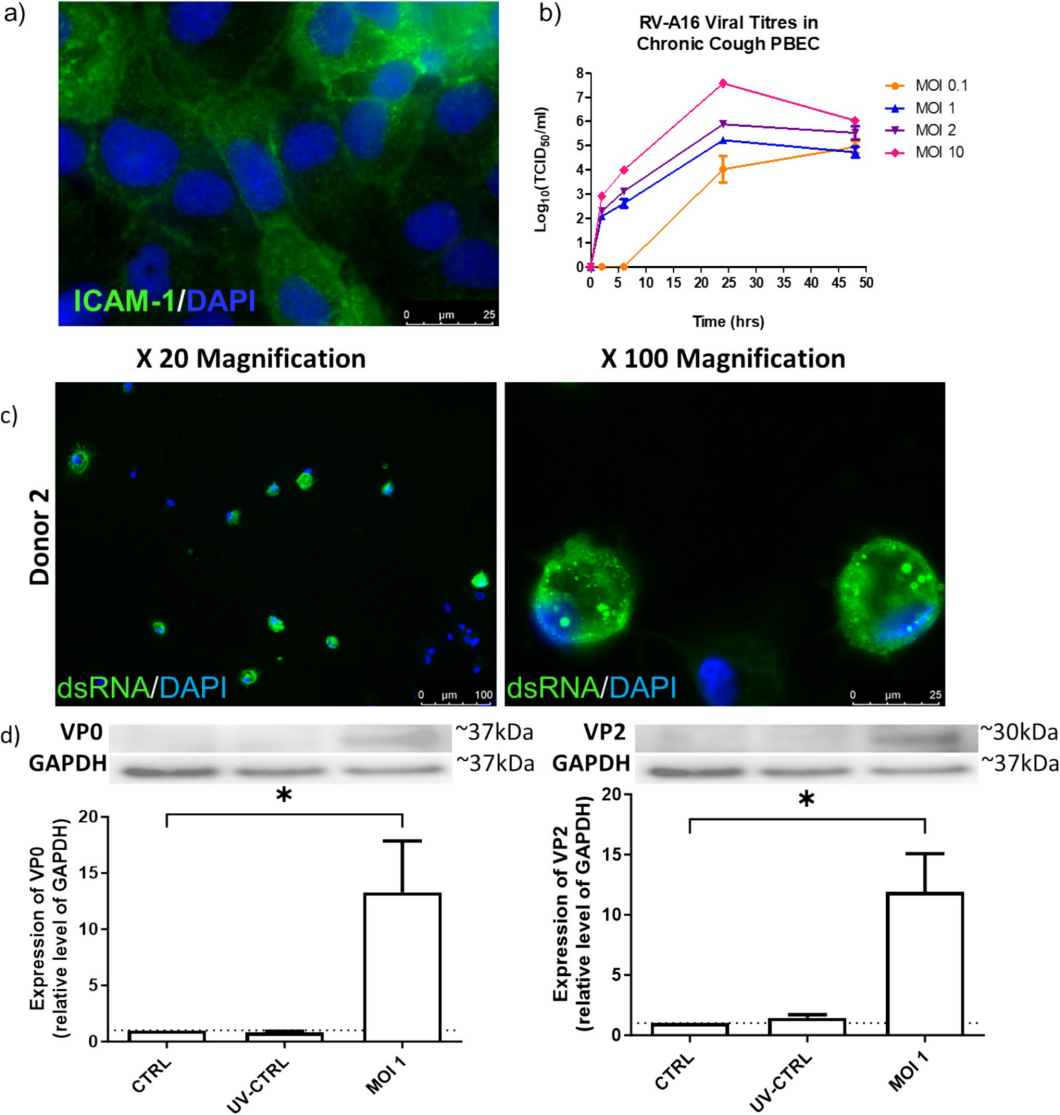
Primary Bronchial Epithelial cells (PBECs) are susceptible to RVA-16 Infection and Permissive for its Reproduction. Immunofluorescence demonstrates protein expression of intracellular adhesion molecule 1 (ICAM-1) in PBECS. Scale bars 25 µm **a**. Viral titrations demonstrate RV-A16 replication in PBEC at MOI 0.1, 1, 2 and 10 over 2 – 48 h.p.i. (PBEC_1). Each point is representative of three independent experiments from one individual donor expressed as mean ± SEM. TCID_50_ = 50% tissue culture infectious dose **b**. Immunofluorescence demonstrates that dsRNA is detected in PBECs infected for 24 h with RV-A16 (MOI 1) at 20 × magnification (scale bar 100 µm) and 100 × magnification (scale bar 25 µm) (PBEC_2) **c**. Representative western blots probed with R16-7 antibody show the presence of viral capsid precursor VP0 (~ 37 kDa) and mature viral capsid protein, VP2 (~ 30 kDa) in PBECs infected with RV-A16 (MOI 1) for 24 h. Semi-quantitative densitometry analysis of immunoreactive bands was carried out and expressed as fold change over GAPDH. Densitometry data are shown as mean ± SEM from three independent experiments (PBEC_2). One way ANOVA with Dunnett’s multiple comparisons test, *P < 0.05 **d**

Similarly to PNEs, PBECs also expressed the ICAM-1, (Fig. 3a, negative controls in supplementary Fig. 2), suggesting that they are susceptible to RV-A16 infection. In addition, viral titrations demonstrated that PBECs were permissive for RV-A16 reproduction, since new infectious viral particles were produced over a 48-h infection period (Fig. 3b). Evidence of viral replication in PBECs was also supported by the specific detection of dsRNA (Fig. 3c, and supplementary Figs. 3 and 5) and the viral capsid proteins VP0 and VP2 (Fig. 3d) in infected PBECs but not in mock-infected cells or cells infected with UV-inactivated virus.

### Direct Rhinovirus Infection Alters the PNE Secretome and Transcriptome

**Fig. 4:**
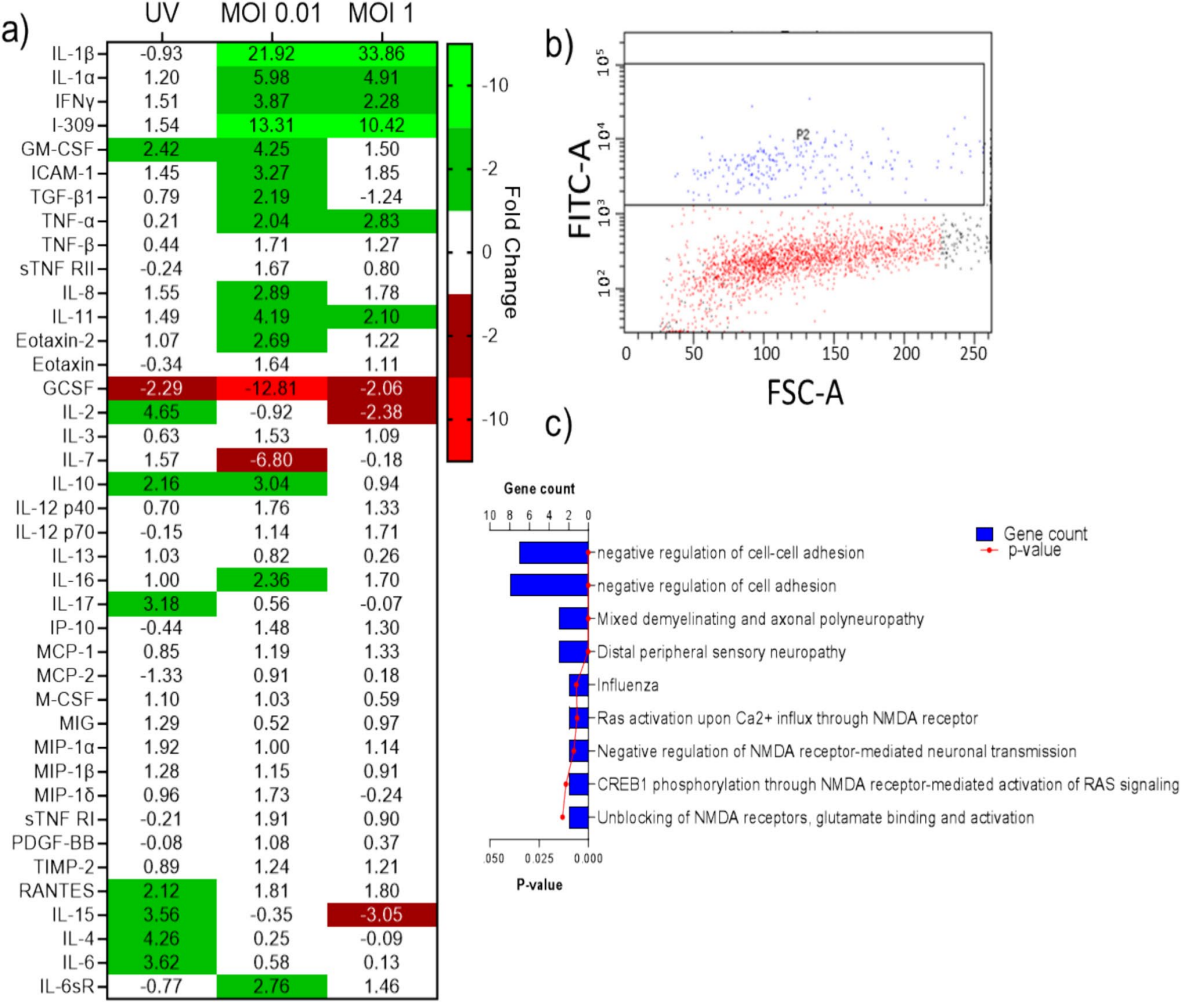
PNE Secretome and Transcriptome are Altered by RVA-16 Infection. Relative fold change in cytokine expression compared to mock-infected PNEs **a**. RV-A16 infected population (dsRNA positive population (P2)) in PNEs is observed using Flow Cytometry. FITC-A = Fluorescein isothiocyanate area, FSC-A = forward-scatter cytogram area **b**. Selected gene ontology terms, human phenotype and diseases terms identified by functional enrichment analysis, or ingenuity pathway analysis in a gene set found to be upregulated in PNEs with RVA-16 infection **c**. Data representative of a single biological replicate (a – c)

Infection of PNEs with RV-A16 at MOI 0.01 or MOI 1 for 24 h resulted in the upregulation (> twofold) of 6 cytokines (IL-1β, IL-1α, IFN-γ, I-309, TNF-α and IL-11) (Fig. 4a). IL-1β was the most upregulated cytokine relative to mock-infected controls at both MOI 0.01 (30-fold) and MOI 1 (34-fold), whilst there was no upregulation in IL-1β in UV infected controls. Flow cytometry analysis indicated that 8.9% of PNEs were dsRNA positive (Fig. 4b), indicative of viral RNA replication. RNA sequencing of this dsRNA positive PNE population revealed the upregulation of the expression of 76 genes compared to mock-infected controls.

Functional enrichment analysis identified multiple gene ontology terms, human phenotype and disease datasets associated with the upregulated genes (selected data presented in Fig. 4c, full data sets presented in supplementary file 1). Gene signatures linked to the negative regulation of cell adhesion (including ABL2, SPINT2, CD164, AKNA, CD74, CBLB, EPCAM, LAPTM5) were identified in the gene ontology category. Additionally, a signature associated with influenza (APOL6, IFIT3) was detected in the disease category. Also of interest, a gene signature for mixed demyelinating and axonal polyneuropathy and distal peripheral sensory neuropathy (MT-ND6, MT-TF, MT-TL1) was present in the human phenotype category. In a similar manner, I.P.A. analysis identified pathways associated with NMDA receptor activity, all sharing the gene CAMK2D (Fig. 4c, full data sets in supplementary file 1).

### Sensitization of PNE TRPA1 Responses by IL-1β Pretreatment

**Fig. 5:**
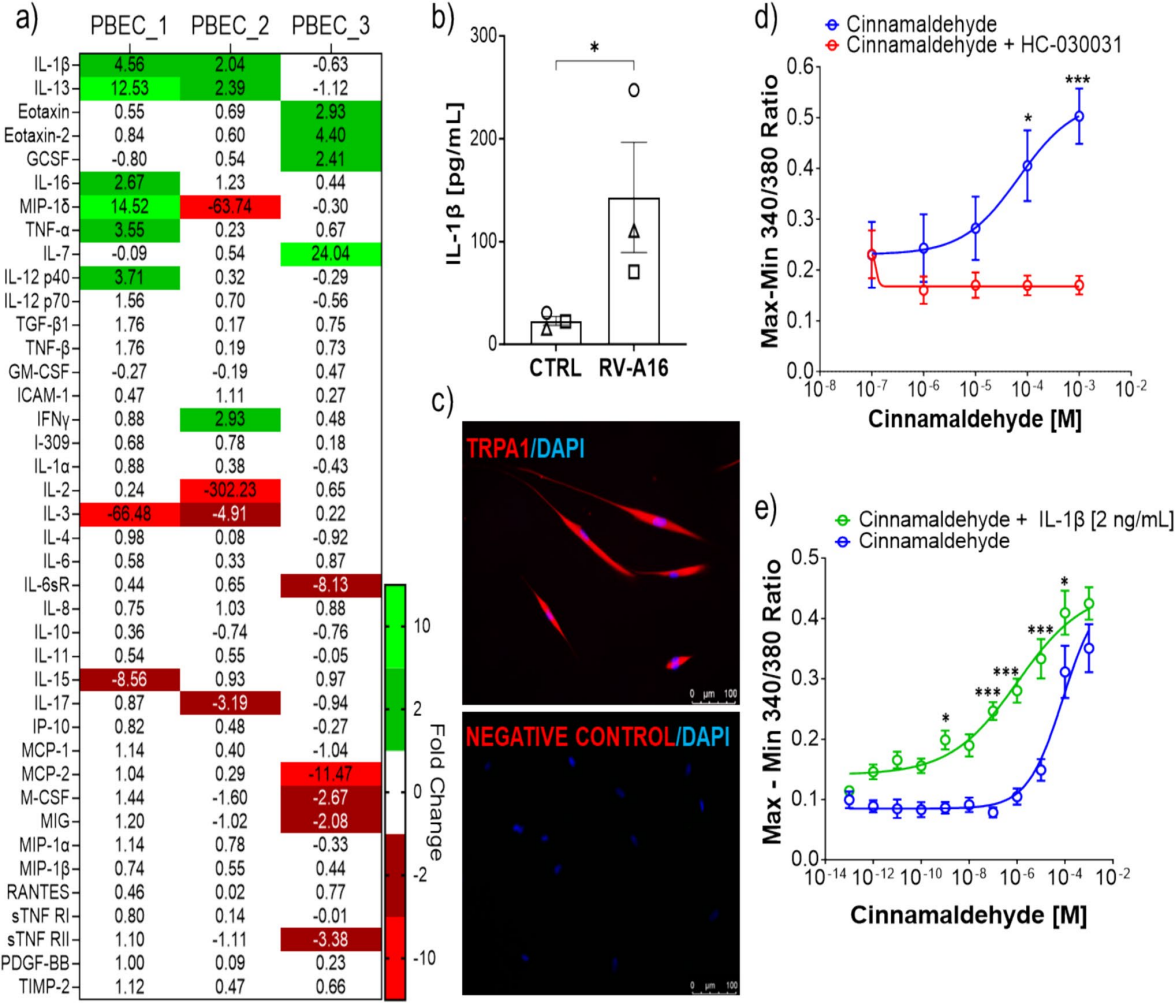
Sensitization of PNE TRPA1 Responses by IL-1β Pretreatment. Relative fold change in cytokine expression in RV-A16 treated PBECs compared to mock-infected control. N = 3 PBEC donors (PBEC_1, PBEC_2 and PBEC_3) **a**. IL-1β release is increased from RV-A16 infected PBECs. Mean with SEM, *N* = 3 PBEC donors (PBEC_2 (○), PBEC_3 (□) and PBEC_4 (∆)). Unpaired T-test, *P < 0.05 **b**. PNEs stained positively for TRPA1, with no extraneous staining observed in primary antibody omitted negative controls. Scale bars 100 µm **c**. Dose–response curve for the TRPA1 agonist cinnamaldehyde [0.0001 to 1 µM] in the absence (blue line) and presence (red line) of the TRPA1 antagonist HC-030031. Each point represents the mean ± SEM from 3–4 independent experiments. Two-way ANOVA with Šídák’s multiple comparisons test, *P < 0.05, ***P < 0.001 **d**. Dose–response curve for the TRPA1 agonist cinnamaldehyde [0.1 pM to 1000 µM] in untreated PNEs (blue line) or PNEs pretreated with IL-1β for 24 h prior to Fura-2AM assay (green line). 4 – 5 independent experiments. Two-way ANOVA with Šídák’s multiple comparisons test, *P < 0.05, ***P < 0.001 **e**

Infection of PBECs with RVA16 at MOI 1 for 48 h.p.i resulted in the upregulation (> twofold) of IL-1β and IL-13 expression in PBEC donor cells (Fig. 5a). Increased IL-1β in the medium of RV-A16 infected PBECs was confirmed by ELISA (*P < 0.05) (Fig. 5b). We then investigated the effect of IL-1β pretreatment on TRPA1 responses in PNEs. Firstly, TRPA1 protein expression was observed in PNEs by immunostaining with no non-specific staining observed in primary antibody omitted negative control (Fig. 5c). Functionality of TRPA1 channels in PNEs was confirmed by calcium mobilisation assay with a dose response relationship observed for cinnamaldehyde (Fig. 5d) with a mean EC_50_ (with 95% confidence interval) of 69 µM (16 µM to 291 µM). TRPA1 responses to 0.1 µM and 1 µM cinnamaldehyde were inhibited by the TRPA1 antagonist HC-030031 (*P < 0.05 & ***P < 0.001) (Fig. 5d). Pretreatment of PNEs with IL-1β resulted in a leftward shift of the TRPA1 dose response curve indicating TRPA1 channel hypersensitivity with a mean EC_50_ (with 95% confidence interval) of 81 µM (30 µM to 881 µM) for untreated PNEs and 1 µM (0.1 µM to 98 µM) for IL-1β treated PNEs (Fig. 5e). Responses to 0.001 µM, 0.1 µM, 1 µM, 10 µM and 100 µM cinnamaldehyde were increased by IL-1β pretreatment (*P < 0.05 & ***P < 0.001) (Fig. 5e).

## Discussion

Using in vitro models, we investigated the effects of RV infection on human neuronal and primary bronchial epithelial cells. We confirmed that the RV entry receptor ICAM-1is expressed on the surface of PNEs and following infection, RV enters the neurons and replicates with an accompanying cytopathic effect and neuroinflammatory response. To investigate the molecular consequences of the direct infection of neurons, we undertook RNA sequencing of PNEs and observed RV-induced expression of genes responsible for antiviral immune responses, maintenance of cell structure and integrity and pathways that may be associated with neuronal sensitisation believed to underpin the clinical features of cough reflex hypersensitivity. We also observed release of inflammatory mediators from virally infected neurons with upregulation in IL-1β, 1–309, IL-1α, IFN-γ and IL-4 when compared with mock-infected and UV-inactivated control samples. Additionally, RV infects and replicates in cultured PBECs, with accompanying release of IL-1β which was subsequently shown to sensitise neurons to the TRPA1 agonist cinnamaldehyde. Taken together these novel findings support both direct and indirect (bystander) consequences of RV infection and its contribution to cough reflex hypersensitivity.

The expression of ICAM-1 on neuronal cells has been reported on cholinergic airway nerves supporting our hypothesis that airway neurons are directly susceptible to infection [23]. IF for dsRNA, generated during viral replication but not naturally present in human cells, was observed in our infected neuronal cells confirming that the virus not only enters the neurons but replicates in them. Flow cytometry indicated approximately 9% of cells expressed dsRNA and were undergoing active virus replication. Importantly, viral titrations demonstrated that PNEs are permissive for RV reproduction, allowing the production of significant amounts of new infectious viral particles (around 4 to 5-log increase in viral titres compared to the start of the infection) over a period of 24 to 72 h and at a range of low MOIs (0.01, 0.1 and 1), considered physiologically relevant [24].

Infected neurons released inflammatory mediators including IL-1β, interferon-gamma (IFN-γ) and IL-4, which are associated with RV infection of the human airways [25–27] and with other neuronal disorders including neuropathic pain and chronic itch [28, 29]. Increased levels of IFN-γ, a cytokine known to induce cough hypersensitivity have been found in the airway of subjects with chronic cough compared to healthy volunteers [26]. We also observed release of the cytokine I-309, also known as Chemokine Ligand 1 (CCL-1), thought to play a role in the development and maintenance of neuropathic pain [30]. Consistent with previous studies [8, 31], our findings demonstrate that neurons infected by respiratory viruses can release inflammatory mediators associated with sensory neuronal dysregulation and disease states, further supporting a role for neuroimmune interactions in viral pathogenesis.

Transcriptomic analysis of infected neurons revealed altered expression of genes related to cell adhesion and structural integrity, consistent with RV-induced cytopathic effects observed in other cell types [32, 33]. We also observed changes in the IFIT3 (Interferon-Induced Protein with Tetratricopeptide Repeats 3) gene involved in the antiviral innate immune response to respiratory viruses including influenza [34]. Interestingly, we observed increased expression of gene signatures linked to neuronal disorders, including distal peripheral sensory neuropathy, supporting the view that chronic cough may have a neuropathic basis [35]. Furthermore, upregulation of cAMP response element-binding protein (CREB1) pathway genes, along with CaMKII-related signalling, are considered important signalling molecules responsible for N-methyl-D-aspartate (NMDA) receptor activation and the development of allodynia, a neuropathic pain state considered similar to cough hypersensitivity [36].

While airway epithelial cells are the primary site for RV replication, we show that RV can also infect and replicate in neuronal cells, triggering neuroinflammatory mediator release. We report that IL-1β, released by both infected PNEs and PBECs, induces heightened sensitivity to the irritant cinnamaldehyde compared to controls suggesting a potential pathway whereby the cough reflex may be sensitised indirectly (in a bystander fashion) following respiratory viral infection.

We acknowledge experimental limitations in that we studied the effect of viral infection on neurons and bronchial epithelial cells in isolation rather than in a more complex array of structural and inflammatory cells that likely interact during viral infection of the human airway. Furthermore, our neuronal model does not entirely replicate the human in vivo circumstance whereby airway neural afferents represent axonal structures projecting from the cell soma (cell bodies) in ganglia located in extra-pulmonary sites. However, it is recognised that viral infection of axonal endings of peripheral neurons is accompanied by anterograde transport of newly synthetised virus to the cell bodies [37, 38]. We believe therefore that our model provides a means to study neuroinflammatory consequences of respiratory viral infection in the human airway.

In conclusion, our data provide evidence for direct and indirect modulation of airway nerves by RV infection. The neuroinflammatory consequences of respiratory viral infection of the human airways has been largely overlooked but does represent a mechanism for accompanying cough reflex hypersensitivity.

## Supplementary Information

Below is the link to the electronic supplementary material.

Supplementary file1 (XLSX 28 KB)

Supplementary file2 (DOCX 3855 KB)

## Data Availability

No datasets were generated or analysed during the current study.
