# Supplementary material for: Neuroinflammatory Consequences of Rhinovirus Infection in Human Epithelial and Neuronal Models
Source: Lung. 2025 Sep 7;203(1):93. doi: 10.1007/s00408-025-00846-y (PMC12414851; doi:10.1007/s00408-025-00846-y)
Supplement: Supplementary file 2 — Supplementary file2 (DOCX 3855 KB) [file 408_2025_846_MOESM2_ESM.docx]

# Online Supplement

## Drugs and Solutions

Radioimmunoprecipitation assay (RIPA) buffer contained 50 mM Trizma® base (Sigma), 150 mM sodium chloride (Thermo Fisher Scientific), 1% NP40 (Sigma-Aldrich), 0.25% sodium deoxycholate (Sigma-Aldrich), 1 mM EDTA (Boehringer Mannhiem) (pH 7.4). Western blot running buffer was prepared by diluting 20x NuPAGE^TM^ MES SDS Running Buffer (Life Technologies), to 1x in deionised water. Western blot transfer buffer was prepared by diluting 20x NuPAGE^TM^ Transfer Buffer (Life Technologies), to 1x in deionised water with 100 mL methanol. Tris-Buffered Saline (TBS) contained 200 mM trizma base (Sigma Aldrich) and 150 Mm sodium chloride (Thermo Fisher Scientific) (pH 7.6). Phosphate buffered saline (PBS) contained 145 mM sodium chloride (Thermo Fisher Scientific), 7.47 mM disodium hydrogen phosphate (Thermo Fisher Scientific) and 2.5 mM sodium phosphate monobasic (Acros Organics, Geel, Belgium) (pH 7.4). Glycine solution was prepared by dissolving glycine powder (VWR Chemicals, Pennsylvania, USA) (MW = 75.07) was dissolved in PBS 1x to make a working concentration of 1 M. 4% paraformaldehyde (PFA) solution contained PFA (Sigma Aldrich) dissolved in distilled water and further diluted in 2X PBS (pH 7.4). Hanks balanced salt solution (HBSS) contained 140 mM sodium chloride (Thermo Fisher Scientific), 5 mM potassium chloride (VWR BDH Chemicals, Leicestershire, UK), 2 mM calcium chloride (Sigma Aldrich), 1 mM magnesium chloride (Sigma Aldrich), 10 mM HEPES free acid (Fluorochem, Glossop, UK) and 5 mM D-glucose (Thermo Fisher Scientific) (pH 7.4).

## Rhinovirus Stock Production and Titrations

The RV strain RV-A16 was obtained from the American Type Culture Collection (ATCC VR-283) and was propagated and titrated in HeLa-H1 (ATCC CRL-1958) cells at 33^o^C. HeLa-H1 cells were infected with the viral stock and incubated at 33^°^C in a 5% CO_2_ atmosphere for 18 hours and then submitted to three freeze thaw cycles. Cell debris were pelleted and virus containing supernatant was aliquoted into single use aliquots. Replication deficient RV-A16 was generated by ultraviolet radiation for 20 minutes and was used to confirm that the effects observed were a result of replicating virus and not a result of passive exposure to the constituents of the virion or the virus culture medium. The inability of the UV-inactivated RV-A16 stock to replicate in HeLa-H1 cells was verified. Viral titres of the RV-A16 stock and of samples collected from PNE or PBEC infections were determined by measuring the 50% tissue culture infectious dose (TCID_50_) in HeLa-H1 cells.

## Viral Titration Assay

A viral titration assay was used to evaluate the production of infectious RV progeny following infection in PNEs or PBECs. PNEs were infected with RV-A16 at MOI 0.01, 0.1 and 1 for 2, 6, 24, 48 or 72 hours. PBEC were cultured to 80% confluency and infected at a range of MOI (0.1, 1, 2 and 10) and timepoints (2, 6, 24 and 48 hours). At the end of the infection period, plates with cells and medium were frozen and thawed 3 times to lyse the cells and release the virus into the medium. Cells were scraped, and the medium and cells were centrifuged to remove cell debris. The supernatant containing the viral particles was titrated by end-point dilution assay, in which HeLa-H1 cells were incubated with 10-fold serial dilutions of virus-containing samples for 5 days at 33^o^C and 5% CO_2_. Cells were assessed by microscopy for the presence or absence of cytopathic effect (CPE) and the viral dilution which caused CPE in 50% of the wells, known as the 50% Tissue Culture Infective Dose (TCID_50_) was calculated and viral titres were expressed as TCID_50_/ml [39].

## Western Blotting

Total cellular extracts were lysed by scraping PNEs or PBECs incubated in RIPA buffer containing Protease-inhibitor cocktail for 15 minutes on ice. Protein quantification of samples was determined using a Pierce^TM^ BCA Protein Assay kit (Thermo Fisher Scientific). Normalised samples for Western blot contained 10 µg of protein, 5 μl of sample buffer (NuPAGE LDS sample buffer (4x), Thermo Fisher Scientific) and 0.5 μl of beta-metacaptoethanol (Sigma Aldrich) adjusted to a total volume of 20 μl using deionised water. Samples were incubated at 70^o^C for 10 minutes to activate the reducing agent prior to loading in NuPAGE 4-12% Bis-Tris gel (Thermo Fisher Scientific) and running at 200V for 40 mins. The gel was transferred to a 0.1 μm Nitrocellulose membrane for at 30 V for 70 mins.

Following transfer, the blot was blocked in 5% BSA (Sigma Aldrich) in TBS containing 0.05% Tween-20 (Sigma Aldrich) at room temperature for 1 hour prior to probing with the R16-7 primary antibody (mab60614, Covalab SAS) at a working concentration of 1:1000 for PNEs and 1:2000 for PBEC. The blot was incubated in the primary antibody overnight at 4^o^C. The blot was incubated with goat anti-mouse IgG peroxidase conjugated to HRP secondary antibody diluted to the working concentration of 1:5000 (Sigma-Aldrich) in the block solution at room temperature for 1 hr. The blot was developed with Clarity Western ECL Substrate (Bio-Rad) and imaged using the Syngene G:Box (230V Model: Chemi XX6, Serial no. DRX/1103) and GynSys application system. Blots were stripped with restore western blot stripping buffer (Thermo Fisher Scientific ) and re-probed with GAPDH antibody at a working dilution of 1:5000 to act as a reference protein for the purposes of band quantification. Blots were analysed using semiquantitative densitometric analysis on ImageJ software (Version 1.41, National Institute of Health, USA)

## Immunostaining

Cells cultured on glass coverslips were fixed with 4% paraformaldehyde (PFA) solution for 10 minutes at room temperature. Residual PFA was quenched with 1 M glycine solution for 10 minutes twice. Permeabilization of cells was carried out with PBS supplemented with 0.05% Triton 100-X for 20 minutes at room temperature. Cells were incubated with 10% normal goat serum (Sigma Aldrich) supplemented with 0.01% Triton 100-X for 1 hour. Cells were incubated with primary antibodies overnight at 4°C (Supplementary Table 2). The following day, cells were incubated with an appropriate secondary antibody for 1 hour at room temperature (Thermo Fisher Scientific). DAPI (4’,6 diamidino-2-phenylindole) (Invitrogen, UK) was used to counterstain nuclei and mount PNEs to microscope slides (Menzel Gläser, Thermo Fisher Scientific). A negative control for each antibody was carried concurrent to the staining procedure with the omission of primary antibodies. Images were acquired using the DM5500 microscope (Leica microsystems).

## Microarray

A commercially available inflammatory antibody array was used to investigate the inflammatory responses of PNEs and PBECs following RV infection (Abcam) according to the manufacturer’s instructions. The array examined the relative expression of 40 analytes; Eotaxin, Eotaxin-2, granulocyte colony stimulating factor (G-CSF), granulocyte-macrophage colony-stimulating factor (GM-CSF), ICAM-1, IFN-γ, I-309, Interleukin (IL)-1α, IL-1β, IL-2, IL-3, IL-4, IL-6, IL-6sR, IL-7, IL-8, IL-10, IL-11, IL-12p40, IL-12p70, IL-13, IL-15, IL-16, IL-17, IP-10, monocyte chemoattractant protein-1 (MCP-1) and -2 (MCP-2), M-CSF, MIG, MIP-1α, MIP-1β, MIP-1δ, regulated upon activation normal T cell expressed and secreted (RANTES), TGF-β1, TNF-α, TNF-β, sTNF RII, platelet-derived growth factor-BB (PDGF BB), TIMP-2.

## ELISA

An IL-1β ELISA (R&D Biosystems) was used to measure IL-1β release from RV infected PBECs.

## Supplementary Table 1 PBEC donor and Phenotype used in the study

| **PBEC Name** | **PBEC Phenotype** | **Gender** | **Age (yrs)** | **FEV_1_ (% predicted)** | **FVC (% predicted)** | **FEV/FVC** | **Pack years** |
| --- | --- | --- | --- | --- | --- | --- | --- |
| PBEC_1 | Refractory chronic cough for 24 years | Female | 68 | 2.41 L (135) | 2.84 L (131) | 85 | 0 - Life time non-smoker |
| PBEC_2 | COPD Heavy smoker | Male | 70 | 2.28 L (83) | 5.26 L (148) | 43 | 40 |
| PBEC_3 | Healthy | Male | 70 | 2.98 L (108) | 4.2 L (116) | 72 | 0- Lifetime non-smoker |
| PBEC_4 | COPD | Female | 55 | 1.03 L (49) | 2.05 L (82) | 50 | 40 |

## Supplementary Table 2 Working concentrations and source of primary antibodies used for immunofluorescence.

| **Name** | **Primary Antibody** | **Secondary Antibody** | **Host**  **Species** | **Supplier (Catalogue No.)** |
| --- | --- | --- | --- | --- |
| TRPA1 | 1:100 | 1:500 | Rabbit | LS Biosciences (LS-B177) |
| FSP | 1:500 | 1:500 | Mouse | Abnova (ab11333-200) |
| PGP9.5 | 1:250 | 1:500 | Mouse | Cedarlane (CL31A3) |
| ICAM-1 | 1:100 | 1:500 | Mouse | Santa Cruz Biotechnology (sc-107) |
| dsRNA | 1:1000 | 1:500 | Mouse | Scicons (RNT-SCI-10010200) |


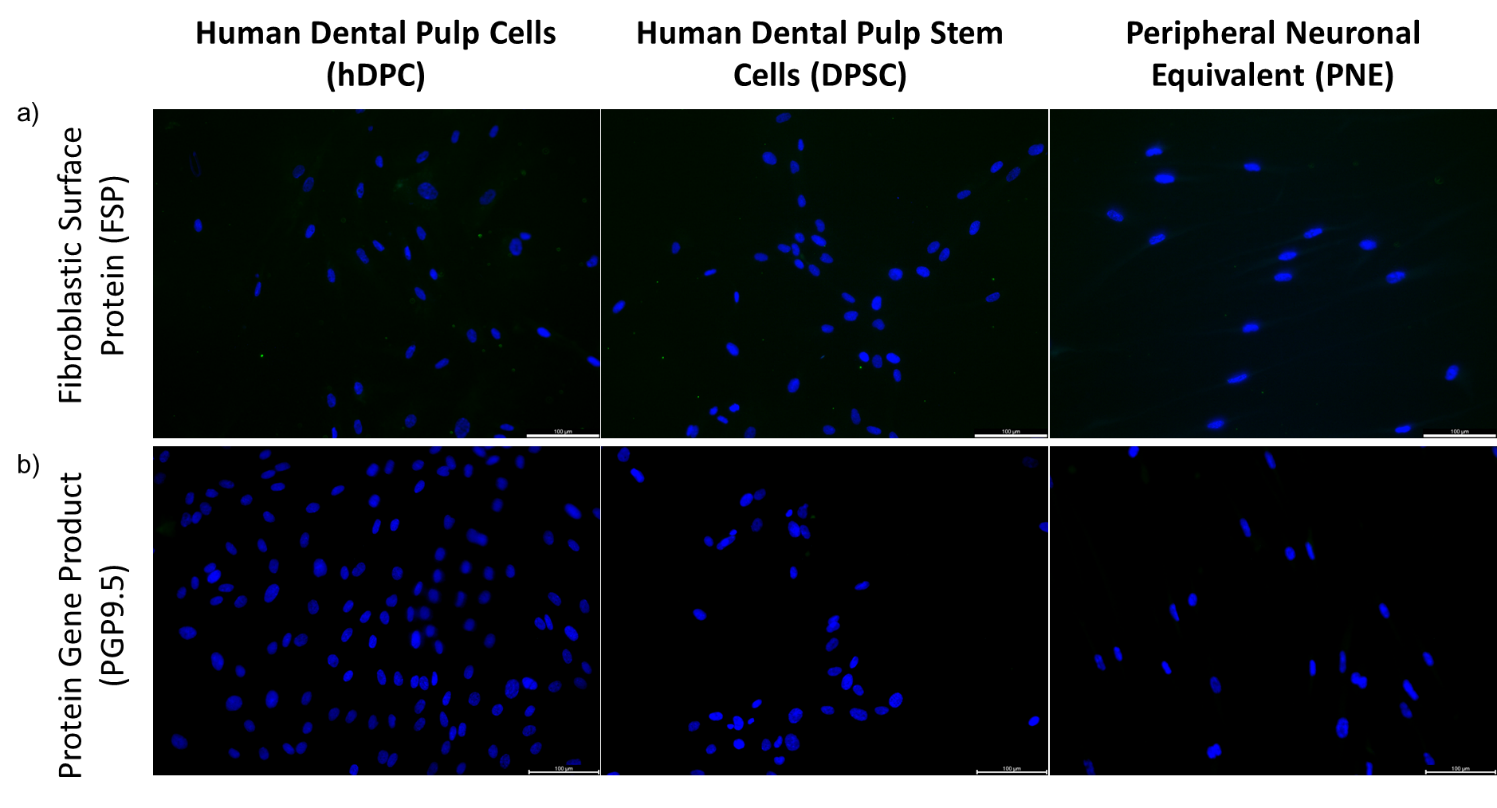


Supplementary Figure 1. Control for PNE Characterisation Staining in Figure 1**.** Primary antibody omitted control for Fibroblastic surface protein (FSP) staining in human dental pulp cells (hDPC), dental pulp stem cells (DPSC) and peripheral nerve equivalents (PNEs) counterstained with DAPI (blue) (a) Primary antibody omitted control for protein gene product (PGP9.5) in hDPC, DPSC and PNEs counterstained with DAPI (blue) (b). Scale bars 100 µm on all images.

**
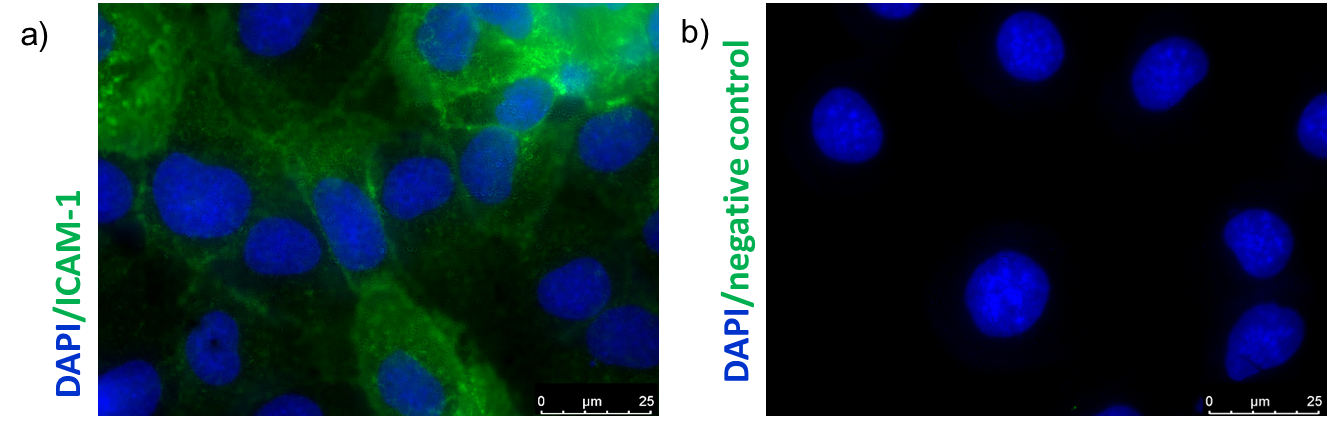
**

**Supplementary Figure 2. Intracellular Adhesion Molecule 1 (ICAM-1) Expression in PBECs.** Immunofluorescence confirms protein expression of ICAM-1 in PBEC (green) and counterstained with DAPI (blue) (a). Primary antibody omitted control for ICAM-1 staining in PBEC and counterstained with DAPI (blue) (b). Images were obtained using a DM5500 microscope. Images acquired using a DM5500 microscope, at 100x objective. Scale bars 25 µm (a & b).


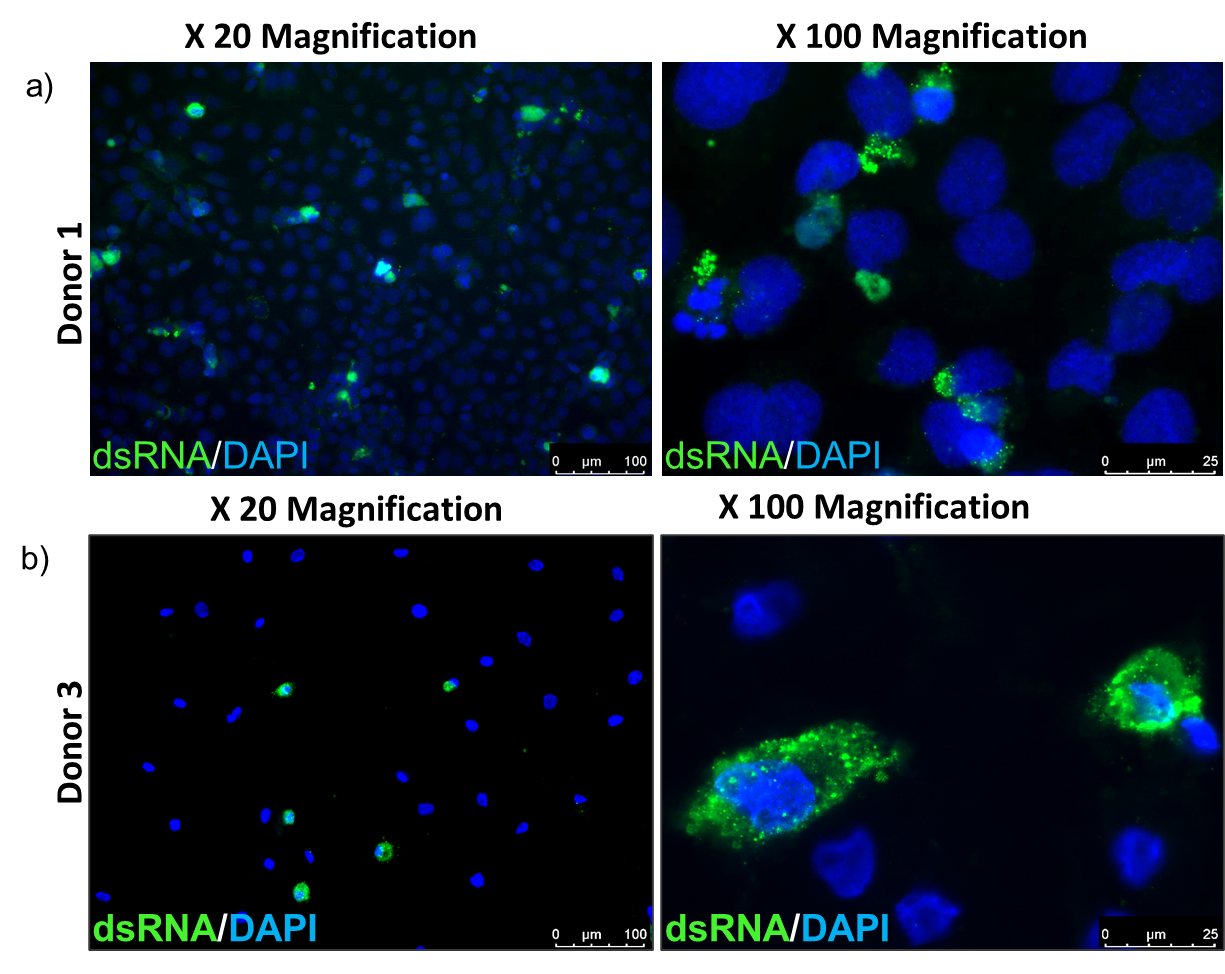


Supplementary Figure 3. **Immunofluorescence Confirms dsRNA is Detected in RV-A16 Infected PBECs.** PBEC_1 (a) or PBEC_3 (b) were infected with RV-A16 at MOI 1 at 24 h.p.i. and stained for dsRNA. Nuclei counterstained with DAPI. Images acquired at 20x magnification with scale bars at 100 μm, or 100x magnification with scale bars at 25 μm (a & b).


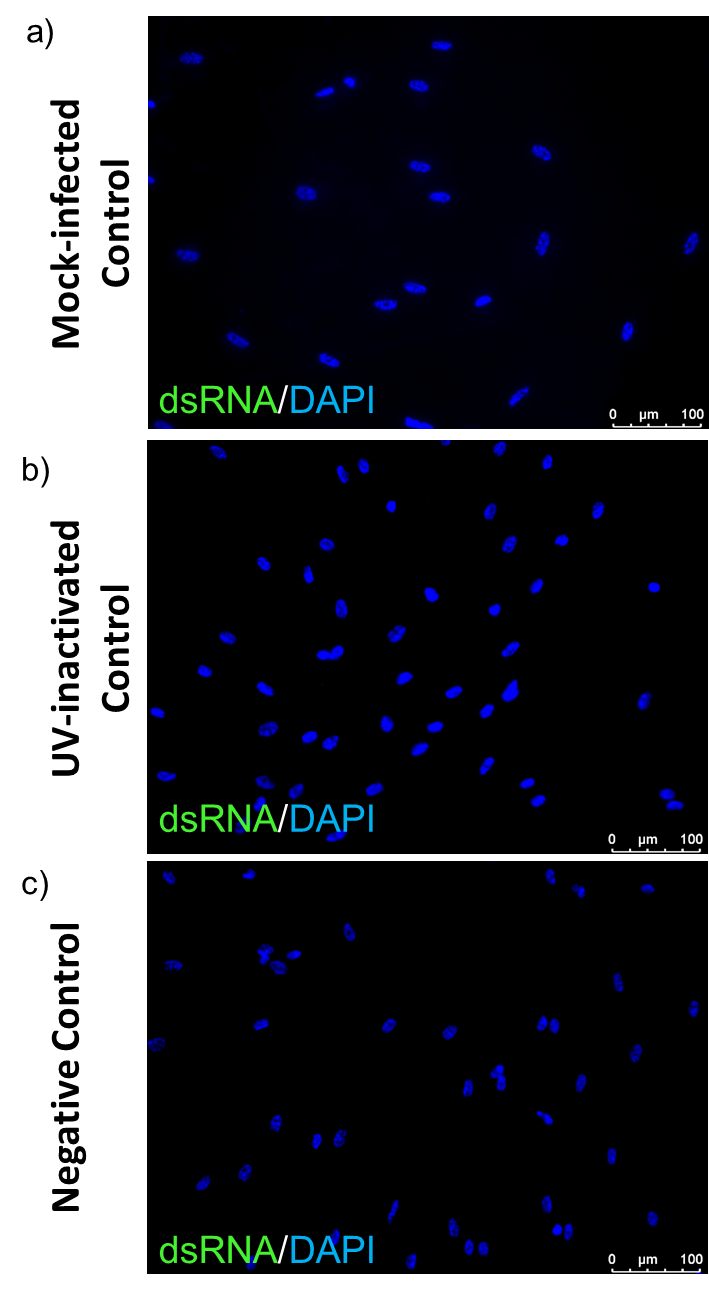


Supplementary Figure 4. Control for PNE dsRNA Immunofluorescence Staining in Figure 2. No dsRNA staining (green) was observed in mock infected PNEs (a), UV-inactivated virus infected PNEs (b), and no extraneous staining was observed in negative primary antibody omitted control PNEs (c). Nuclei counterstained with DAPI. Images acquired at 20x magnification with scale bars at 100 μm (a – c).


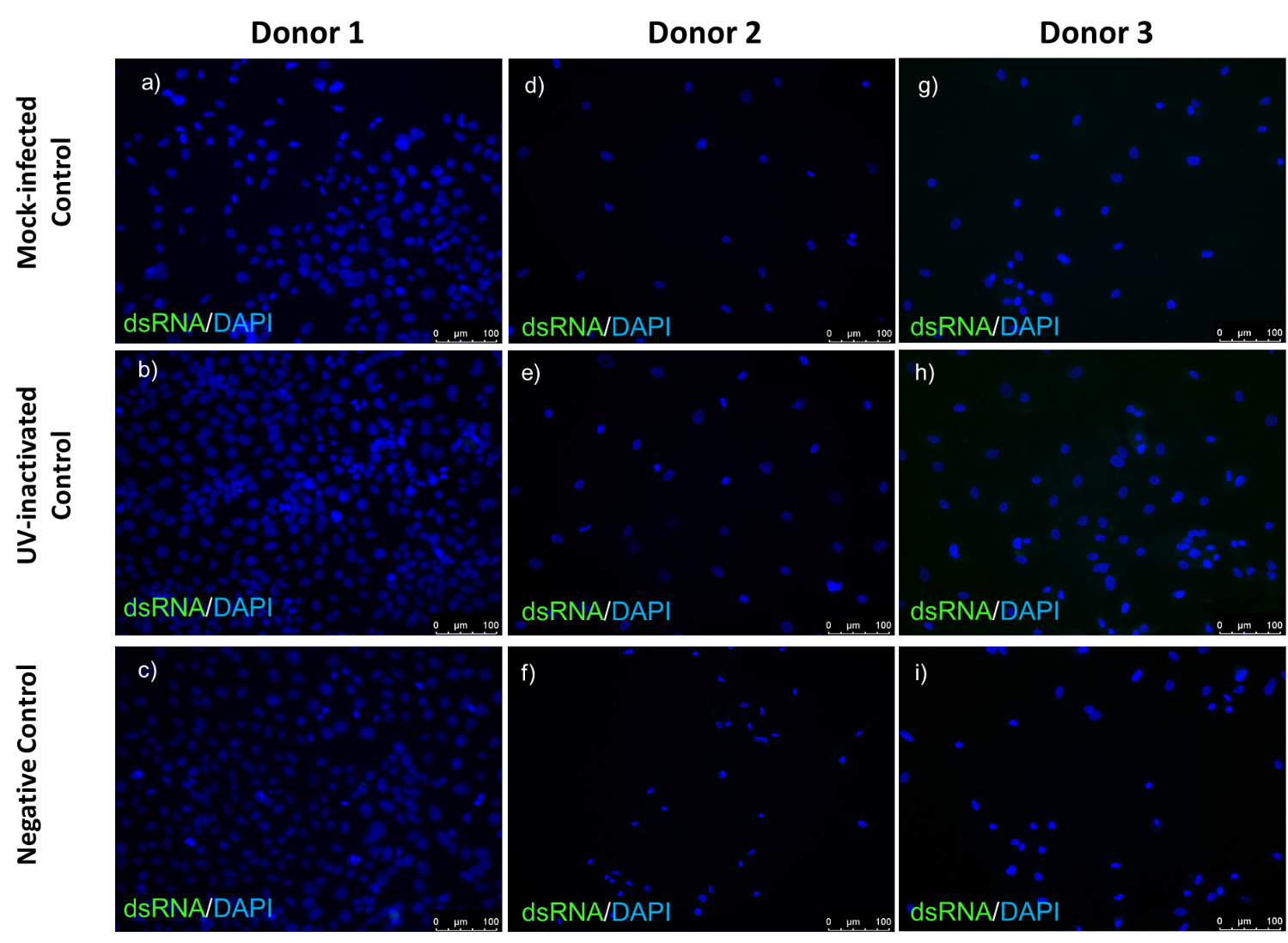


Supplementary Figure 5. Control for PBEC dsRNA Immunofluorescence Staining in Figure 3 & Supplementary Figure 3**.** No dsRNA staining (green) was observed in mock infected PBECs (a), UV-inactivated virus infected PBECs (b), and no extraneous staining was observed in negative primary antibody omitted control PBECs (c) for PBEC_1. No dsRNA staining (green) was observed in mock infected PBECs (d), UV-inactivated virus infected PBECs (e), and no extraneous staining was observed in negative primary antibody omitted control PBECs (f) for PBEC_2. No dsRNA staining (green) was observed in mock infected PBECs (g), UV-inactivated virus infected PBECs (h), and no extraneous staining was observed in negative primary antibody omitted control PBECs (i) for PBEC_3. Nuclei counterstained with DAPI. Images acquired at 20x magnification with scale bars at 100 μm (a – i).
